# Viscoelastic testing for hepatic surgery: a systematic review with meta-analysis—a protocol

**DOI:** 10.1186/s13643-016-0326-1

**Published:** 2016-09-06

**Authors:** Kate Elizabeth McCrossin, David Edmund Piers Bramley, Elizabeth Hessian, Evelyn Hutcheon, Georgina Imberger

**Affiliations:** 1 Department of Anaesthesia, Royal Brisbane and Women’s Hospital, Butterfield Street, Herston, QLD 4006 Australia; 2 Department of Anaesthesia and Pain Medicine, Western Health, Gordon Street, Footscray, VIC 3011 Australia; 3 Western Health Library Service, Western Health, Gordon Street, Footscray, VIC 3011 Australia

**Keywords:** Hepatic, Viscoelastic testing, TEG, ROTEM, Surgery, Anaesthesia

## Abstract

**Background:**

Viscoelastic tests, including thromboelastography (TEG) and rotational thromboelastometry (ROTEM), provide a global assessment of haemostatic function at the point of care. The use of a TEG or ROTEM system to guide blood product administration has been shown in some surgical settings to reduce transfusion requirements. The aim of this review is to evaluate all published evidence regarding viscoelastic testing in the setting of hepatic surgery.

**Methods:**

We will search MEDLINE, EMBASE and the Cochrane Central Register of Controlled Trials databases to identify randomised controlled trials examining the use of viscoelastic testing for hepatic surgery. Two reviewers will independently screen titles and abstracts of studies identified and will independently extract data. Any disagreements will be resolved by discussion with a third reviewer. A meta-analysis will be conducted if feasible.

**Discussion:**

Viscoelastic devices such as TEG and ROTEM are increasingly available to clinicians as a bedside test. Patients undergoing hepatic surgery have a significant risk of blood loss and coagulopathy requiring transfusion. Theoretical benefits of use of a TEG or ROTEM system in the hepatic surgical setting include a rationalisation of blood products, a reduction in transfusion-related side effects, an improvement in patient outcomes including mortality, and a reduction in cost. This systematic review will summarise the current evidence regarding the use of viscoelastic testing for hepatic surgery.

**Systematic review registration:**

PROSPERO CRD42016036732

**Electronic supplementary material:**

The online version of this article (doi:10.1186/s13643-016-0326-1) contains supplementary material, which is available to authorized users.

## Background

Thromboelastography (TEG) and rotational thromboelastometry (ROTEM), also known as viscoelastic tests, are point of care coagulation tests that provide a global assessment of haemostatic function. Viscoelastic tests monitor whole-blood coagulation, evaluating clot formation, strength, and lysis or retraction [1], and are the only tests that can provide a rapid diagnosis of hyperfibrinolysis [2]. Performed at the bedside or in the operating theatre by clinical staff, TEG and ROTEM are convenient and provide immediate results.

Point of care coagulation testing allows clinicians to administer the specific blood products required to replace deficient components of the coagulation system. TEG- or ROTEM-guided transfusion algorithms have been shown to reduce the number of cardiac surgical patients requiring blood transfusion [3]. Viscoelastic testing may also improve blood product management, reduce blood product transfusion and reduce transfusion-related complications [4]. These clinical benefits may also be cost beneficial in select patient groups [5].

Clinical benefit of the use of TEG or ROTEM has been suggested in settings including cardiac surgery [3, 5], trauma-induced coagulopathy [6] and post-partum haemorrhage [7]. Convincing systematic review of the evidence is, however, lacking. In a systematic review looking at TEG and ROTEM in the context of massive transfusion in 2013, Afshari et al. found only one eligible trial with a low risk of bias and concluded that there was an absence of evidence to assess the effect on morbidity and mortality [3]. The following year, in the setting of critically ill patients, Haas et al. found only four small randomised controlled trials [7].

Despite a lack of systematic evidence, there are strong theoretical arguments for the benefit of TEG and ROTEM. These arguments have particular strength in the setting of surgery involving the liver. Despite improvements in surgical technique, extensive blood loss in liver resections may still require blood product transfusion, increasing rates of perioperative morbidity and mortality [8]. Viscoelastic testing, which helps guide transfusion decisions, may lead to improved patient outcomes including mortality, rationalised use of blood products and reduced cost. This systematic review aims to evaluate all published evidence from randomised controlled trials regarding viscoelastic testing in the setting of hepatic surgery.

## Methods

### Objective

The purpose of this study is to systematically review the current available evidence regarding the use of viscoelastic testing in adults undergoing hepatic surgery.

### Protocol and registration

We will follow the methodological standards outlined in the *Cochrane Handbook for Systematic Reviews of Interventions* [9]. The findings will be reported as guided by the Preferred Reporting Items for Systematic Reviews and Meta-Analyses (PRISMA) [10] (also see Additional file 1 ‘PRISMA checklist for submission’). The review has been prospectively registered with the International Prospective Register of Systematic Reviews (PROSPERO) [11].

### Eligibility criteria

Table 1 shows eligibility criteria for population, intervention, comparison and study design. Table 2 shows eligibility criteria for outcomes. We will not apply language restrictions. Studies will be included independent of publication status and publication date.

**Table 1:** Eligibility criteria for population, intervention, comparison and study design

Study characteristics	Inclusion criteria	Exclusion criteria
Patient population	Adults ≥18 years undergoing hepatic surgery: - Elective surgery involving resection of hepatic tissue - Hepatic transplantation	
Intervention	Use of viscoelastic testing (TEG or ROTEM), either alone or within an algorithm	
Comparison	Standard care for that trial setting, where standard care does not include viscoelastic testing	
Study design	Randomised controlled trials	Randomised trials with a cross-over design Cluster randomised trials Case control studies Cohort studies Case series/case reports

**Table 2:** Eligibility criteria for outcomes

Outcome	Criteria	Data type	Planned summary measure
Primary outcome	1. 30-day mortality (all-cause) Using mortality data in each eligible trial that is less than and closest to 30 days	Categorical	Relative risk
Secondary outcomes	1. Long-term mortality Using mortality data in each eligible trial that is >30 days and the longest follow-up time for that trial	Categorical	Relative risk
	2. Blood loss (mL)—included if measured from the start of surgery, defined a priori and measured equally in both groups. We will use follow-up that is closest to including 24 h after the end of surgery.	Numerical	Standardised mean difference
	Number of participants receiving blood products—included if measured from the start of surgery, defined a priori and measured equally in both groups. We will use follow-up that is closest to including 24 h after the end of surgery.	Categorical	Relative risk
	3. Any type of blood product		
	4. Autologous red blood cells		
	5. Any type of autologous coagulation factor		
	6. Autologous fresh frozen plasma		
	7. Autologous cryoprecipitate		
	8. Autologous platelets		
	Volume of blood products administered—included if measured from the start of surgery, defined a priori and measured equally in both groups. We will use follow-up that is closest to including 24 h after the end of surgery.	Numerical	Standardised mean difference
	9. Red blood cells (mLs or units)		
	10. Fresh frozen plasma (mLs or units)		
	11. Cryoprecipitate (mLs or units)		
	12. Platelets (mLs or units)		
	Use of other pro-coagulant interventions—included if measured from the start of surgery, defined a priori and measured equally in both groups. We will use follow-up that is closest to including 24 h after the end of surgery.	Categorical	Relative risk
	13. Tranexamic acid		
	14. Recombinant Factor VIIa		
	15. Other recombinant factor concentrates		
	16. Serious complications associated with blood loss and blood product transfusion—included if measured from the start of surgery, defined a priori and measured equally in both groups. We will use follow-up that is closest to including 24 h after the end of surgery. - Unplanned intensive care unit (ICU) admission <24 h - Myocardial infarction (MI) <7 days - Cardiac arrest <24 h - Central nervous system complications – Cerebrovascular accident (CVA) or traumatic brain injury (TBI) <7 days post-operation - renal complications <7 days—where a clear definition of renal complications has been provided in the paper and measured equally in both groups	Categorical	Relative risk
	17. Thromboembolic complications—included if measured from the start of surgery, defined a priori and measured equally in both groups. We will use follow-up that is closest to including 7 days after the end of surgery. - New arterial or deep venous thrombosis - Pulmonary embolism	Categorical	Relative risk
	18. Cost—we will include any cost outcomes from studies where a numerical cost outcome has been clearly defined and equally measured for both groups.	Numerical	Standardised mean difference

We will include data for the following outcomes if they were specifically measured in both groups and defined a priori

### Information sources and search

We will search the following databases: MEDLINE, EMBASE and the Cochrane Central Register of Controlled Trials databases. The Appendix shows the search strategy which will be used for each database. References of all included trials will be scanned for eligibility.

### Study selection

Two reviewers (KM and GI) will independently screen titles and abstracts of studies identified via the search method as outlined in the Appendix. We will discuss any discrepancies and will retrieve full copies of all potentially relevant studies. Two reviewers (KM and GI) will then independently assess the complete publications for eligibility. Any disagreements will be resolved by discussion with a third reviewer (DB or EH). We will use Covidence software to coordinate the study selection process [12].

### Data collection process and data items

Two reviewers will independently extract the data outlined below (KM and GI). Any disagreements will be resolved by discussion with a third reviewer (DB or EH). We will contact authors to retrieve any missing data. In particular, we anticipate finding trials where the population is broader than our inclusion criteria but includes patients undergoing hepatic surgery as a subgroup. When outcome data for hepatic surgery patients are not presented for this subgroup in the publication, we will contact authors and attempt to retrieve these data.

For all studies, we will extract the following data:

1. Study characteristics—first author, year of publication, funding source, study design and setting, duration of follow-up, number randomised.
2. Population characteristics—inclusion and exclusion criteria, patient characteristics, underlying disease or condition (including bleeding diatheses and anticoagulant/anti-platelet drugs), presence and severity of liver disease, other co-morbidities, type and duration of surgery and type of anaesthesia.
3. Intervention characteristics—the type of viscoelastic test, whether a transfusion algorithm was used, which transfusion algorithm was used
4. Comparison intervention characteristics—performance of coagulation studies, or no coagulation testing.
5. Outcomes—as described in Table 2; number analysed for each outcome, number of drop-outs with reason. In the case of multi-arm trials, we will include all arms that fulfil our defined criteria for the intervention in our intervention arm and all arms that fulfil our defined criteria for the control in our control arm.

### Risk of bias in individual studies

We will assess the risk of bias according to guidelines in The Cochrane Handbook of Systematic Reviews [9]. For each included trial, we will assess selection bias (random sequence generation and allocation concealment), performance bias (blinding of participants and personnel), detection bias (blinding of outcome assessment), attrition bias (incomplete outcome data), reporting bias (selective reporting) and any other sources of bias.

When all risk of bias parameters are assessed as satisfactory, the trial will be categorised as low risk of bias. Our assessment of low risk of bias will be for the lowest possible risk. Given that blinding of the testing for the treating physician is impossible, the lack of this blinding will not preclude an assessment of satisfactory.

### Synthesis of results

We will provide a descriptive synthesis of the findings of our review. If there are two or more eligible studies, reporting on the same outcome and with low risk of bias, meta-analysis will be performed. All testing will be two-sided. We will assess statistical heterogeneity using the chi-squared test (Cochran’s Q), the *I*-squared statistic [13] and diversity (*D*^2^) [14]. Irrespective of statistical estimation of heterogeneity, we will use a random effects model (Mantel-Haenszel method) [15] to perform the meta-analyses. A sensitivity analysis will be performed using a fixed effect model, and we will report any relevant differences. Continuity correction will be used for zero event trials [16].

The risk of random errors, caused by sparse data and repetitive testing, will be assessed using Trial Sequential Analysis (TSA) [14, 17–19]. We will estimate a diversity-adjusted required information size [14] using 0.05 for type 1 error, 0.2 for type 2 error, control event proportions estimated from the un-weighted mean of the proportions in the control groups in the included trials and a relative risk reduction (RRR) of 20 %. Additional sensitivity analyses will be conducted for each TSA using the empirical RRR to estimate effect size.

We will use Review Manager software for the conventional meta-analyses [20]. TSA software will be used for the TSA [21]. We will use R statistical software for any other analyses or calculations required [22], and all code will be published to ensure reproducibility of all our analyses.

### Subgroup analysis

For our primary outcome, mortality, and for our secondary outcomes, our first analyses will include all populations of patients undergoing hepatic surgery.

When possible, we will then conduct two subgroup analyses based on hepatic surgery types:

1. Elective operations involving hepatic resection
2. Hepatic transplantation

For each of the above analyses, when possible, we will do two further subgroup analyses based on population:

1. Severe hepatic failure
2. Pre-existing coagulopathy

We will accept any reasonable definitions for the above subgroups provided by the individual trials, as long as they have been clearly defined a priori and where the outcomes have been measured in these subgroups equally in both arms of the trial.

### Risk of bias across studies

If more than 10 trials are included in the cumulative meta-analyses, we will assess the risk of publication bias by Funnel plots and Egger’s/Begg’s test [23].

### Grading the quality of the evidence

We will consider the quality of the evidence using the GRADE Working Group’s guidelines [24]. We will provide a summary of the evidence, based on the GRADE format, for every outcome that we meta-analyse. For each included outcome, we will consider the risk of bias in included trials, the risk of publication bias, the precision, the consistency and the directness.

## Discussion

As the technology simplifies and becomes more cost-effective, viscoelastic devices such as TEG and ROTEM are increasingly available to clinicians as a bedside test. The most recent incarnations of both systems, the TEG6s and the ROTEM Sigma, are portable and easy to use, with reduced blood handling compared with previous systems [25, 26]. These systems are point-of-care, producing a shorter turnaround time compared with traditional coagulation testing.

Arguably, the most significant advantage over traditional testing is that viscoelastic tests evaluate the function rather than the quantity of individual haemostatic factors [27]. By recording the process of clot formation as a trace, both TEG and ROTEM display parameters that indicate the need for specific corrective blood components [1]. During haemorrhage with resulting coagulopathy, these results can be used to guide an individualised and timely response.

Due to an increased risk of preoperative coagulopathy associated with liver dysfunction and to the surgical risks associated with surgery involving the liver, patients undergoing hepatic surgery have a significant risk of perioperative blood loss and coagulopathy requiring transfusion. Functional coagulation in the presence of liver disease can be poorly predicted by conventional coagulation testing [28]. TEG and ROTEM may therefore offer particular advantages for this patient population, by allowing more timely and potentially more accurate decisions about whether to give coagulation products and which to give. These decisions may result in better clinical outcomes, including mortality. With more timely and targeted treatment of coagulopathy in the setting of hepatic surgery, blood loss may be reduced, avoiding both the adverse clinical outcomes associated with blood loss itself and with red cell transfusion [29]. Moreover, timing and targeted treatment of coagulopathy may also be associated with a reduced use of coagulation products themselves, also leading to a reduction in the adverse clinical outcomes associated with these transfusions. If the use of ROTEM or TEG in the setting of hepatic surgery does lead to a reduction in transfusion, and is associated with improved clinical outcomes, the potential cost benefit of using these tests represents a further important advantage.

We aim to systematically examine the effects of TEG or ROTEM in the setting of hepatic surgery. The *Cochrane Handbook* recommends considering inclusion of clinically important outcomes as primary in a systematic review, such as all-cause mortality [9]. In the case of TEG and ROTEM in the setting of hepatic surgery, the important clinical question is how do these tests, in association with whatever clinical intervention results from using them, alter clinical outcomes. This systematic review will summarise the available evidence regarding the use of viscoelastic testing for hepatic surgery, focusing on mortality as a primary outcome and also exploring the effect on blood loss, blood product use, adverse effects of blood loss and transfusion, and cost.

## Appendix

Search strategies

Search strategies finalised 24/6/16

Medline (Ovid)

1. exp Liver/
2. (hepatic or liver or hepato*).mp.
3. 1 or 2
4. exp General Surgery/
5. (surger* or surgical* or reconstruct* or resection* or segment* or transplant*).mp.
6. exp Anesthesia, General/
7. (anaesthesia or anesthesia).mp.
8. 4 or 5 or 6 or 7
9. 3 and 8
10. “blood transfusion*”.mp.
11. hemorrhage*.mp.
12. exp Carcinoma, Hepatocellular/su [Surgery]
13. exp Liver Neoplasms/su [Surgery]
14. exp Hepatectomy/
15. hepatectom*.mp.
16. “hemi hepatectom*”.mp.
17. hemi-hepatectom*.mp.
18. exp Liver Transplantation/
19. “liver transplant*”.mp.
20. 9 or 10 or 11 or 12 or 13 or 14 or 15 or 16 or 17 or 18 or 19
21. (viscoelastic adj3 (test* or device* or method*)).mp.
22. exp Thrombelastography/
23. thromboelasto*.mp.
24. thrombelasto*.mp.
25. thromb?elastograph*.mp.
26. TEG.mp.
27. ROTEM.mp.
28. ((“point of care” or POC or POCT or test* or function* or therap* or analys*) adj4 coag*).mp.
29. 21 or 22 or 23 or 24 or 25 or 26 or 27 or 28
30. 20 and 29
31. randomized controlled trial.pt.
32. controlled clinical trial.pt.
33. randomized.ab,ti.
34. placebo.ab,ti.
35. randomly.ab,ti.
36. trial.ab,ti.
37. groups.ab,ti.
38. 31 or 32 or 33 or 34 or 35 or 36 or 37
39. 30 and 38
40. (animals not (humans and animals)).sh.
41. 39 not 40

Embase (Ovid)

1. Exp Liver
2. (hepatic or liver or hepato*).mp.
3. 1 or 2
4. exp general surgery/
5. (surger* or surgical* or reconstruct* or resection* or segment* or transplant*).mp.
6. exp general anesthesia/
7. (anaesthesia or anesthesia).mp.
8. 4 or 5 or 6 or 7
9. 3 and 8
10. “blood transfusion*”.mp.
11. hemorrhage*.mp.
12. exp liver cell carcinoma/su [Surgery]
13. exp liver tumor/su [Surgery]
14. exp liver resection/
15. hepatectom*.mp.
16. “hemi hepatectom*”.mp.
17. hemi-hepatectom*.mp.
18. exp liver transplantation/
19. “liver transplant*”.mp.
20. 9 or 10 or 11 or 12 or 13 or 14 or 15 or 16 or 17 or 18 or 19
21. (viscoelastic adj3 (test* or device* or method*)).mp.
22. exp thromboelastography/
23. thromboelasto*.mp.
24. thrombelasto*.mp.
25. thromb?elastograph*.mp.
26. TEG.mp.
27. ROTEM.mp.
28. ((“point of care” or POC or POCT or test* or function* or therap* or analys*) adj4 coag*).mp.
29. 21 or 22 or 23 or 24 or 25 or 26 or 27 or 28
30. 20 and 29
31. randomized controlled trial.pt.
32. controlled clinical trial.pt.
33. randomized.ab,ti.
34. placebo.ab,ti.
35. randomly.ab,ti.
36. trial.ab,ti.
37. groups.ab,ti.
38. 31 or 32 or 33 or 34 or 35 or 36 or 37
39. animals/ not humans/
40. 38 not 39
41. 30 and 40

Cochrane

1. MeSH descriptor: [Liver] explode all trees
2. hepatic or liver or hepato*:ti,ab,kw (Word variations have been searched)
3. #1 or #2
4. MeSH descriptor: [General Surgery] explode all trees
5. surger* or surgical* or reconstruct* or resection* or segment* or transplant*:ti,ab,kw (Word variations have been searched)
6. MeSH descriptor: [Anesthesia, General] explode all trees
7. anaesthesia or anesthesia:ti,ab,kw (Word variations have been searched)
8. 4 or 5 or 6 or 7
9. #3 and #8
10. “blood transfusion*”:ti,ab,kw (Word variations have been searched)
11. hemorrhage*:ti,ab,kw (Word variations have been searched)
12. MeSH descriptor: [Carcinoma, Hepatocellular] explode all trees and with qualifier(s): [Surgery – SU]
13. MeSH descriptor: [Liver Neoplasms] explode all trees and with qualifier(s): [Surgery – SU]
14. MeSH descriptor: [Hepatectomy] explode all trees
15. Hepatectom*:ti,ab,kw (Word variations have been searched)
16. “hemi hepatectom*:ti,ab,kw (Word variations have been searched)
17. hemi-hepatectom*:ti,ab,kw (Word variations have been searched)
18. MeSH descriptor: [Liver Transplantation] explode all trees
19. “liver transplant*:ti,ab,kw (Word variations have been searched)
20. #9 or #10 or #11 or #12 or #13 or #14 or #15 or #16 or #17 or #18 or #19
21. Viscoelastic near/3 (test* or device* or method*):ti,ab,kw (Word variations have been searched)
22. Thromboelasto* or thrombelasto*:ti,ab,kw (Word variations have been searched)
23. Thromb?elastograph*:ti,ab,kw (Word variations have been searched)
24. TEG:ti,ab,kw (Word variations have been searched)
25. ROTEM:ti,ab,kw (Word variations have been searched)
26. (“point of care” or POC or POCT or test* or function* or therap* or analys*) near/4 coag*:ti,ab,kw (Word variations have been searched)
27. #21 or #22 or #23 or #24 or #25 or #26
28. 20 and 27

## Additional file

Additional file 1: PRISMA checklist for submission. (DOCX 17 kb)
